# RNAi-Based Approaches to Control Mycotoxin Producers: Challenges and Perspectives

**DOI:** 10.3390/jof10100682

**Published:** 2024-09-29

**Authors:** Alexander A. Stakheev, Michael Taliansky, Natalia O. Kalinina, Sergey K. Zavriev

**Affiliations:** 1 M.M. Shemyakin and Yu.A. Ovchinnikov Institute of Bioorganic Chemistry, 117997 Moscow, Russia; 2 A.N. Belozersky Institute of Physico-Chemical Biology, Lomonosov Moscow State University, 119992 Moscow, Russia

**Keywords:** RNA interference, double-stranded RNAs, plant protection, mycotoxins, *Fusarium*, *Aspergillus*, HIGS, SIGS

## Abstract

Mycotoxin contamination of food and feed is a worldwide problem that needs to be addressed with highly efficient and biologically safe techniques. RNA interference (RNAi) is a natural mechanism playing an important role in different processes in eukaryotes, including the regulation of gene expression, maintenance of genome stability, protection against viruses and others. Recently, RNAi-based techniques have been widely applied for the purposes of food safety and management of plant diseases, including those caused by mycotoxin-producing fungi. In this review, we summarize the current state-of-the-art RNAi-based approaches for reducing the aggressiveness of key toxigenic fungal pathogens and mycotoxin contamination of grain and its products. The ways of improving RNAi efficiency for plant protection and future perspectives of this technique, including progress in methods of double-stranded RNA production and its delivery to the target cells, are also discussed.

## 1. Introduction

Fungal diseases are among the most significant factors of agricultural losses and food security threats worldwide [1,2]. Climate change along with the development of transport and trade lead to the emergence of new pathogenic species as well as expansion of the area of existing ones. Besides direct impacts on quality and quantity of yields, fungi of genus *Fusarium*, *Aspergillus*, *Penicillium* and *Alternaria* are able to produce a wide range of toxic secondary metabolites known as mycotoxins. The ability to synthesize toxic secondary metabolites was acquired by different fungal taxa during evolution [3] and became an important tool for occupying novel ecological niches [4]. Nowadays, the number of known mycotoxins is estimated as 300–400 [5], but the most widely distributed and important among them are aflatoxins, trichothecenes, fumonisins and zearalenone. These compounds are highly toxic to mammals, possessing carcinogenic, teratogenic, hepatotoxic and immunosuppressive properties, inhibiting protein biosynthesis and causing mitochondrial dysfunction [6,7,8,9,10,11]. Moreover, trichothecenes, especially deoxynivalenol (DON) and toxins of the recently described NX group [12], act as a virulence factor in host plants, facilitating tissue colonization and inhibiting plant immune response [13,14].

The toxic effect of mycotoxins, as well as direct economic impacts caused by their producers, makes it necessary to develop efficient methods to control toxigenic fungi in agricultural plants. Traditional approaches, including crop rotation or developing resistant cultivars, are time-consuming and unable to prevent mycotoxin accumulation completely. The application of fungicides has been the most widely applied technique to control mycotoxigenic fungi [15]. However, this approach has several disadvantages, including possible induction of mycotoxin synthesis [16,17], negative effects on human and animal health [18] and impact on biodiversity [19]. The development of pathogens’ resistance is another serious challenge, reducing the efficiency of this approach for combating toxigenic fungi [20,21]. The use of mycotoxin biosynthesis inhibitors of natural origin has been a promising alternative to fungicides, mainly in terms of biological safety [22,23,24,25], but it is often difficult to isolate an individual active compound from a total extract and to obtain it in quantitates appropriate for massive use.

Recently, attention has been paid to methods based on the control of gene expression via RNA-interference (RNAi). After the first observation in petunia plants [26], RNAi was described in many other organisms of different taxa. Generally, RNAi represents a conserved mechanism, the central role in which is played by small RNAs (sRNAs), able to regulate gene expression at the post-transcriptional level (post-transcriptional gene silencing, PTGS) [27]. RNAi is involved in various cellular processes, such as maintenance of genome stability, adaptation to stressful conditions, chromatin modification, protection against viruses and alien nucleic acids, development and pathogenesis [28,29,30,31,32,33,34].

Romano and Machino [35] first demonstrated that transformation of the fungus *Neurospora crassa* with different portions of two carotenogenic genes (*al-1* and *al-3*) resulted in phenotypic changes. Later, Fire et al. [36] showed that injection of specific exogenous dsRNA into the nematode *Caenorhabditis elegans* led to gene-specific silencing. These discoveries have opened up an opportunity of using RNAi as an efficient biotechnological tool. Particularly, it has been used for analyses of plant and fungal gene functions, as well as for improving traits of industrially, agronomically and medically important fungal strains [37,38,39]. Since the 2000s, RNAi-based approaches have been applied for solving different problems of plant protection, including management of mycotoxins and their producers. In this review, we discuss what has been undertaken in the field of application of RNAi to control mycotoxigenic fungi and what should be conducted to bring this approach to widescale use in agricultural practice. 

## 2. RNAi Principles and Its Current Applications

### 2.1. Mechanism of RNAi and Key Components of the Process

The main players of RNAi processes in fungi, as well as in other eukaryotes, are proteins of Dicer and Argonaute families, as well as RNA-dependent RNA-polymerases (RdRps) [40,41,42]. In brief, the RNAi pathway includes two steps. In the first step, the trigger RNA (either dsRNA or hairpin RNA) is processed into small interfering RNAs (siRNAs), a class of sRNAs, typically 21–25 base pairs in length containing 3′ dinucleotide overhangs. This process is catalyzed by Dicer or Dicer-like (DCLs) enzymes, members of the RNase III family. The siRNAs are then amplified by RdRPs. siRNAs produced by Dicer cleavage and those synthesized by RdRPs are called primary and secondary siRNAs, respectively [43]. In the second step, siRNAs are loaded into the Argonaute (AGO) protein to form the RNA-induced silencing complex (RISC). Then, one of the siRNA strands (passenger) is removed from the RISC and another (guide) one directs RISC to the mRNA target to cleave it by the AGO protein [44,45,46,47].

Despite the fact that RNAi is a highly conserved mechanism, fungal species differ by the number of RNAi machinery components they possess. Several species, such as *Saccharomyces cerevisiae* and *Ustilago maydis*, do not have DCLs and AGOs since they were lost during evolution [48,49,50]. However, the latter species is able to produce sRNAs from tRNAs and 5.8 S ribosomal RNA [51]. Dicer-independent mechanisms of RNAi were also identified in some other fungi [28,52]. *F. graminearum*, one of the most widespread and devastating toxigenic species, possesses two DCLs, two AGOs and five RdRps. According to Chen et al. [45], only *Fg*AGO1 and *Fg*DCL2 are necessary for silencing. Later, Zeng et al. [53] and Gaffar et al. [54] demonstrated that *Fg*AGO2 and *Fg*DCL1 participate in sexual ascosporogenesis and pathogenic processes, while *Fg*AGO1 and *Fg*DCL2 regulate asexual conidia formation and germination. *F. graminearum* RdRps were shown to be responsible for conidiation [54], but their role in the production of secondary siRNAs has been unclear. According to Song et al. [55], *F. asiaticum*, a member of the *Fusarium graminearum* species complex (FGSC), lacks the ability to produce secondary siRNAs and this results in short-time silencing of a target gene. This inability can be considered as a serious potential obstacle for applying RNAi to manage fungal pathogens in the field.

### 2.2. Cross-Kingdom RNAi and dsRNA Uptake

It was recently described that sRNAs can bi-directionally move between plants and pathogenic organisms, being an important element of an arms race between them. This phenomenon is called ‘cross-kingdom RNA interference’ (ckRNAi) [56,57,58]. Pathogens send sRNAs to the host cells to suppress plant immunity, while plants use this mechanism to inhibit pest virulence [59,60,61,62,63]. Interestingly, sRNAs produced by a fungal pathogen often affect target gene(s) expression via the host plant’s RNAi machinery. For instance, Weiberg et al. [59] demonstrated the presence of *Botrytis cinerea*-produced sRNAs associated with AGO1 of *Arabidopsis thaliana*. sRNAs of a vascular pathogen *F. oxysporum* f.sp. *lycopersici* bind to tomato Argonaute 4a (SlyAGO4a) [64]. Toxigenic *F. graminearum* was shown to send sRNAs, targeting the resistance-related *CEBiP* gene, into the cells of common wheat [65]. Mechanisms of ckRNAi are far from being fully understood; however, some of them have been elucidated. Plants can translocate sRNAs by cell-to-cell mechanism via the plasmodesmata (short-range transfer) or via the vascular system, predominantly phloem (long-range movement) [66,67]. Recent studies found that plants synthesize extracellular vesicles (EVs) to carry RNA molecules and other effectors directed against pathogenic fungi [62,68,69]. In turn, many fungal species release EVs to deliver RNAs into host plant cells [70]. Surprisingly, apart from sRNAs, full-length mRNAs can also be transported into target cells and translated [71]. On the other hand, several reports suggest that EVs play a small role or no role in sRNAs movement, suggesting the existence of other mechanisms of secretion, movement and maintaining the stability of sRNAs [72,73,74]. 

The ability to uptake dsRNA varies among fungal species. Several fungi, such as *Botrytis cinerea*, *Sclerotinia sclerotiorum*, *Rhizoctonia solani*, *Aspergillus niger* and *Verticillium dahliae*, as well as some *Fusarium* spp., demonstrated a high rate of dsRNA uptake [75,76,77]. At the same time, *Trichoderma virens* and the oomycete *Phytophthora infestans* had limited uptake; *Colletotrichum gloeosporioides* and *Zymoseptoria tritici* showed no uptake at all [75,78]. It is believed that uptake efficiency is related to fungal lifestyle and that necrotrophic fungi are more susceptible to dsRNA application. Mechanisms of uptake are still unknown for most fungal species; however, it was demonstrated that clathrin-mediated endocytosis, a well-known eukaryotic mechanism, is involved in dsRNA uptake in *S. sclerotiorum* [79]. In this study, live cell imaging experiments showed that hyphal tips of younger, rapidly growing hyphae of *S. sclerotiorum* are responsible for most active uptake processes. Several proteins, including vacuolar ATPase (VATPase), the heavy chain of the clathrin complex (CHC), clathrin adaptor protein 2 complex (AP2), ADP ribosylation factor-like 1 protein (Arf72A) and amphiphysin (Amph) were shown to be essential for successful dsRNA uptake. At the same time, specific receptors responsible for dsRNA binding have yet to be identified.

### 2.3. HIGS and SIGS

The very first studies on the use of RNAi to control mycotoxin production were carried out by integration of siRNAs-encoding sequences into the genomes of toxigenic strains [80,81] followed by in vitro and in planta tests of mycotoxin accumulation inhibition. These studies demonstrated the principal possibility of the use of RNAi to inhibit mycotoxin biosynthesis and fungal growth. In addition, they allowed uncovering functions of some genes and their opportunity to be used as targets for RNAi. At the same time, further development of RNAi-based techniques as tools for pathogen management in the field has led to the emergence of such approaches, as host-induced gene silencing (HIGS) and spray-induced gene silencing (SIGS).

**Figure 1:**
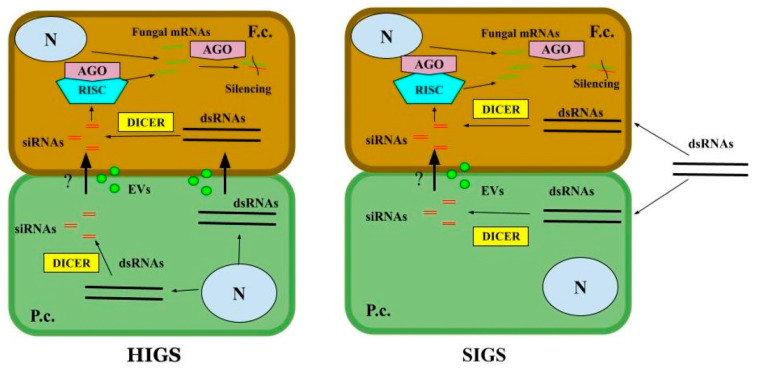
A schematic representation of HIGS (**left**) and SIGS (**right**) mechanisms. HIGS: a cell of a transgenic plant generates sequence-specific dsRNAs to target corresponding fungal genes. These dsRNAs are both transferred to fungal cells by extracellular vesicles or other mechanisms and cleaved into siRNAs by plant DCL proteins. dsRNAs transferred to fungal cells also cleaved into siRNAs by fungal DCL proteins. siRNAs are loaded into the AGO protein to form the RNA-induced silencing complex (RISC). Then, the one of the siRNA (passenger) strand is removed from the RISC and another (guide) one directs RISC to the mRNA target to cleave it. SIGS: The dsRNAs are sprayed onto the plant surface. One part of dsRNAs are taken by the fungus and then produced into siRNAs by the fungal RNAi machinery. Another part of dsRNAs is taken by the plant cells and produced into siRNAs by plant RNAi machinery and then transferred into fungal cells. The following processes are analogous to those of HIGS. P.c.—plant cell; F.c.—fungal cell; N—nucleus; EVs—extracellular vesicles.

In HIGS, the natural mechanism of ckRNAi is used for plant protection against pathogenic organisms by expressing exogenous dsRNAs homologous to target pathogen’s gene(s). A simplified scheme of HIGS is shown in Figure 1. To perform HIGS, a dsRNA or a hairpin RNA-encoding construct that targets a specific pathogen’s gene is transformed into the host plant [82]. The first successful application of HIGS against toxigenic fungus was described in 2010 for tobacco plants, expressing a hairpin β-glucuronidase (*GUS*) gene-targeted dsRNA to inhibit *F. verticillioides* [83]. Since then, a number of studies have been carried out demonstrating the applicability of this approach to inhibit different toxigenic species and corresponding toxins. HIGS has been successfully used to protect wheat [84,85], maize [86,87,88,89,90,91], barley [92,93], Arabidopsis [92], peanut [94] and groundnut [95] (summarized in Table 1). However, this technique has several shortcomings that make it necessary to search for improvements and possible alternatives. Transgenic approaches are time-consuming and technically sophisticated. The application of HIGS requires transformation of the corresponding host plant, though there are still no transformation protocols for many crops. The key methods used for plant transformation are microprojectille bombardment, *Agrobacterium* infiltration and transgenesis, but all these techniques have specific limitations and drawbacks [96]. It is anticipated that the introduction of the CRISPR/Cas9 system will facilitate plant genome engineering [97,98], but to date several challenges remain. A principal obstacle of the wider use of HIGS is that the legislation of GMO is country-specific and there are many countries where these organisms are prohibited to develop and use even for scientific purposes [99]. Another method technically similar to HIGS is VIGS (virus-induced gene silencing) which represents a virus vector-based transient synthesis of dsRNA in a host plant [100]. The most often-used virus vector for VIGS is barley stripe mosaic virus (BSMV) [101]. BSMV-based VIGS was successfully used to protect wheat against toxigenic *F. culmorum* [85,102] and several non-toxigenic fungi, such as *Blumeria graminis* and *Puccinia striiformis* [82,101,103]. Obvious restrictions of this approach are methodological difficulties of efficient plant infection and possible entry of the virus into the environment. As an alternative to the use of BSMV, Zhang et al. [104] described the application of *F. graminearum* gemytripvirus (FgGMTV1)-based vector to convert an *F. graminearum* strain into hypovirulent ones by silencing two pathogenicity-related genes *TRI101* and *FgPP1*, and therefore, to protect wheat against infection. The authors showed that the obtained hypovirulent strains can be used as biocontrol agents themselves, inhibiting fungal infection and decreasing DON content after wheat spikes being infected by these strains’ mycelia together with conidial suspension of virulent *F. graminearum* strain PH-1. Mycovirus-based VIGS seems to be promising to manage mycotoxin producers, including under field conditions. However, mycoviruses is a relatively poor-studied group of viruses; thus, corresponding vectors able to mediate efficient silencing have yet to be developed.

**Figure 2:**
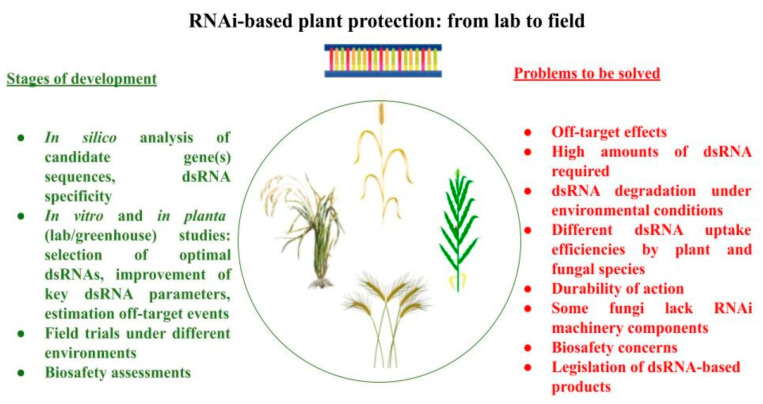
Key stages of experimental adaptation of RNAi-based products to field applications and main problems to be solved on the way.

SIGS is based on the application of specific dsRNA (s) directly to the pathogen or plant surface, generally using a foliar spray. A simplified scheme of SIGS is shown in Figure 1. Koch et al. [76] described the first successful use of SIGS to control mycotoxigenic fungi. A long-coding dsRNA (CYP3dsRNA), targeting three cytochrome P450 lanosterol C-14α-demethylases of *F. graminearum*, was applied at Arabidopsis and barley leaves by spraying. Six days after the inoculation, the development of brownish lesions on dsRNA-treated leaves was significantly lower than in control samples and the expression levels of three target genes were reduced in the range of 48% to 58%. Moreover, the authors demonstrated that silencing and inhibition of fungal infection were observed in distant (non-treated) parts of barley leaves, indicating dsRNA translocation within the plant as well as the fact that fungal, not the plant’s, RNAi machinery plays the key role in the silencing process. Later, the movement of sprayed dsRNA from leaves to stems and roots of barley during three days after treatment was confirmed by Biedenkopf et al. [105]. The direct comparison of SIGS and HIGS methods, performed by Koch et al. [93] using the aforementioned *CYP51*-based system demonstrated that the growth inhibition of *F. graminearum* on barley leaves was significantly higher for SIGS in comparison to HIGS (in lab conditions). For instance, infected leaf areas were reduced by 7% (HIGS) and 80% (SIGS) when CYP51A-dsRNA is applied. At the same time, there is a lack of studies, comparing HIGS vs. SIGS efficiencies both in vitro and *in planta*. By now, SIGS has proven to be effective in protecting wheat [77,102,106,107,108] and barley [109] against toxigenic *Fusarium*. Therefore, SIGS-based approaches have great prospects, but there are several serious limitations, hindering the possibility of massive use of these techniques. These limitations are summarized in Figure 2 and discussed below.

**Table 1:** Summary of HIGS and SIGS assays to control toxigenic fungal species described to date.

Fungal Species	Method(s)	Host Plant(s)	Target Gene(s)	Effect on Mycotoxin Production	Reference
*F. graminearum*	SIGS	Barley	*CYP51A* + *CYP51B* + *CYP51C*	-	[76]
*F. asiaticum*	SIGS	Wheat	*Myo5* (myosin 5)	-	[77]
*F. verticillioides*	HIGS	Tobacco	*GUS* (β-glucuronidase)	-	[83]
*F. graminearum*	HIGS	Wheat	*Chs3b* (chitin synthase 3b)	78–85% reduction (DON)	[84]
*F. culmorum*	HIGS, VIGS	Wheat	*FcFg1* (secreted lipase); *FcFmk1* (MAP-kinase); *FcGls1* (β-1,3-Glucan synthase); *FcChsV* (chitin synthase V)	-	[85]
*A. flavus*	HIGS	Maize	*Amy1* (alpha-amylase)	Drastically reduced (AFB1, AFB2)	[86]
*A. flavus*	HIGS	Maize	*AFLR*	14-fold reduction (AFB1, AFB2)	[87]
*A. flavus*	HIGS	Maize	*Alk* (alkaline protease)	84–87% reduction (aflatoxin)	[88]
*A. flavus*	HIGS	Maize	*AFLM* (versicolorin dehydrogenase)	54.2–95.3% reduction (AFB1)	[89]
*A. flavus*	HIGS	Maize	*p2c* (polygalacturonase)	30.3–93.7% reduction (aflatoxin)	[90]
*A. flavus*, *A. parasiticus*	HIGS	Maize	*AFLC*	100% reduction (AFB1)	[91]
*F. graminearum*	HIGS	Arabidopsis, barley	*CYP51A* + *CYP51B* + *CYP51C* (sterol 14α-demethylase; single product)	-	[92]
*F. graminearum*	HIGS, SIGS	Arabidopsis, barley	*CYP51A*; *CYP51B*; *CYP51C*	-	[93]
*A. flavus*	HIGS	Peanut	*AFLS* (transcriptional regulator) + *AFLR* (transcriptional regulator) + *AFLC* (polyketide synthase) + *Pes1* (nonribosomal peptide synthetase) + *AFLep* (efflux pump)	100% reduction (AFB1, AFB2)	[94]
*A. flavus*	HIGS	Groundnut	*NsdC* (Cys_2_His_2_ zinc finger transcriptional regulator) + *VeA* (development and secondary metabolism regulator) + *AFLR* + *AFLM*	>1000-fold reduction (AFB1)	[95]
*F. culmorum*	SIGS, VIGS	Wheat	*TRI5* (trichodiene synthase)	53–85% reduction (DON, in vitro)	[102]
*F. asiaticum*	SIGS	Wheat	*β2-tubulin*	-	[106]
*F. graminearum*	SIGS	Wheat	*TRI6* (transcriptional regulator)	72% reduction (DON)	[107]
*F. graminearum*	SIGS	Wheat	Chs7 (chitin synthase 7); Gls (glucan synthase); Pkc (protein kinase C)	25–50% reduction (DON)	[108]
*F. graminearum*	SIGS	Barley	*AGO* (ARGONAUTE); *DCL* (DICER)	-	[109]

-: mycotoxin content was not estimated; AFB1/2: aflatoxins B1/B2.

## 3. The Ways to Improve RNAi Efficiency and Facilitate Its Transition to the Field-Scale Application

### 3.1. Selection of Target Gene(s)

The selection of a target gene is crucial for the successful application of RNAi-based disease control. An ideal RNAi gene target must be essential for survival and pathogenicity and should not have functional redundancy [110]. Generally, the potential target genes for mycotoxin producers can be divided into the following groups: (i) genes involved in mycotoxin biosynthesis or regulating it; (ii) genes encoding global regulation factors; and (iii) genes encoding fungal virulence factors. The first group includes the genes of the trichothecene biosynthetic cluster in *Fusarium* spp., such as *TRI5* and *TRI6* [102,107], or different genes of the aflatoxin biosynthetic cluster in *Aspergillus* spp. [87,89,91,94]. These genes are specific for these mycotoxin producers; therefore, their use should reduce the risk of unintended silencing of other targets (off-target effects, OTEs) [111]. On the other hand, these genes can be more polymorphic even at the intraspecific level than housekeeping genes [112] and this fact can potentially reduce the efficiency of RNAi by a higher number of mismatches. Moreover, a fungal isolate may be able to produce different types of mycotoxins, thus silencing of a specific cluster gene does not inhibit the production of other toxic metabolites. A possible alternative is to use genes encoding global transcription factors that regulate different fungal processes. An example of such a gene is *Vel*, responsible for growth, hyphal development, conidia formation and secondary metabolite production in *Fusarium*. This gene, as well as its homolog *veA*, was successfully used to control non-toxigenic *F. oxysporum* f. sp. *cubense* [113] and *A. flavus* [95], respectively. Potential target genes related to fungal virulence include those encoding enzymes and other factors that interfere with plant immunity and can therefore facilitate the infection process. Among these are *Amy1*, *Alk* and *p2c* genes applied to protect maize against *A. flavus* [86,88,90] and chitin synthases that proved effective against *F. graminearum* and *F. culmorum* infection in wheat [84,85,108]. Several studies have shown the great potential of using genes targeted by the main fungicides, such as azoles [76,77,92,93,106]. The genes encoding key components of fungal RNAi machinery (AGOs and DCLs) have also been effective as targets for silencing [109]. In some of the aforementioned studies, the authors silenced multiple genes at once. This approach looks promising in terms of efficiency and reliability, although it can be limited because of higher costs and off-target risks. In further studies, it would be interesting to expand the range of regulatory genes; firstly, all those encoding global regulators, participating in both developmental processes and mycotoxin production. In addition, some groups of genes involved in toxin synthesis, including those that participate in zearalenone biosynthesis in *Fusarium* spp., have not been tested. Finally, little is known regarding the real efficiency of different targets under field conditions.

### 3.2. Design and Optimization of dsRNAs 

One of the most challenging tasks that any RNAi-based study faces is potential OTEs. An example of difficulties caused by the off-target effect is described by Masanga et al. [87]. In this study, host-induced silencing of gene encoding transcriptional factor *AFLR* in *A. flavus* led to simultaneous lowering of aflatoxin content (14-fold), stunting and reduced kernel placement in the transgenic maize. This observation was attributed to the silencing of unintended genes in transformed plants by *AFLR*-targeted siRNAs. A genome-wide bioinformatics analysis is still the best tool to find possible off-target sequences. The ability of the widely used Basic Local Alignment Search Tool (BLAST, [114]) algorithm to predict local alignments of short sequences is limited [115], but some alternative bioinformatics tools, such as dsCheck [116], ERNAi [117], GESS [118], si-RNA finder [119], pssRNAit [120] and SeedMatchR [121] were developed. The remaining challenge is that a great number of plant, fungal and bacterial genomes are still to be sequenced. Nevertheless, several recommendations to minimize off-target effects can be proposed. For instance, long GC-rich fragments as well as those containing ≥16 nt-long sequences that are complementary to any non-target mRNA should be avoided [122,123]. According to Chen et al. 2024 [124], the critical sequence identity of off-target effects is approximately 80%. Another possible problem is that dsRNAs produced from different regions of the same gene often demonstrate different silencing efficiencies. For instance, Johnson et al. [125] showed that targeting different segments of *FUM1* and *FUM8* genes of *F. verticillioides* led to different rates of fumonisin B1 synthesis inhibition. Similar results were obtained by Song et al. [77] for five different regions of *F. asiaticum MYO5* gene. Baldwin et al. [126] demonstrated that targeting both long (~600 nt) and short (250–300 nt) regions of *TRI6* gene of *F. graminearum* resulted in repeatable patterns of the produced siRNAs. Unequal distribution of siRNAs derived from fragments of five aflatoxin biosynthetic genes of *A. flavus* was later shown by Power et al. [127]. Moreover, the authors elucidated that most of siRNAs possess uracil as the 5′ terminal base and cytosine as the 3′ terminal base. These observations can be explained by the specificities of DCL processing, AGO binding and amplification of dsRNA by RdRps [128,129,130]. Today, there are no algorithms and software for accurately predicting profiles of siRNAs, derived from a certain long dsRNA. Therefore, in vitro and in vivo testing of dsRNAs, targeting different segments of the same gene(s) followed by a selection of the most efficient candidate is still an unavoidable stage of any RNAi experiment.

Selecting an optimal concentration of dsRNA for SIGS is another important task, influencing the silencing efficiency and potential off-target effects. It is believed that the concentration should be determined for an individual pathogen and that the dsRNA dose that proved effective in vitro might not show the same efficiency in open air. To date, there is a lack of studies comparing the effectiveness of different dsRNA concentrations to protect plants against mycotoxin producers. Tretiakova et al. [102] demonstrated that the strongest effect on DON accumulation was obtained using 0.96 µg of dsRNA complementary to the *TRI5* gene of *F. culmorum* as well as that higher concentrations were not more efficient. A dsRNA length is another important factor of designing an optimal assay. As in the case of optimal dsRNA concentrations, it seems to be adopted for a specific pathogen and genes to be silenced. Several studies [76,126] demonstrated that longer dsRNAs (500–600 nt) are more efficient than shorter variants. On the other hand, Höfle et al. [131] demonstrated that SIGS-based efficiencies decreased from 80% for 200–500 nt constructs to 65% for 800 nt constructs and to 50% for >1500 nt dsRNA constructs. Moreover, Tretiakova et al. [102] used a short 161 nt dsRNA to successfully silence *TRI5* gene expression. As mentioned above, the silencing efficiency of dsRNAs is determined by their nucleotide sequences rather than molecules’ length *per se*. In theory, shorter molecules that are efficiently processed by DCL should be more promising for RNAi than longer ones giving low siRNAs yield. On the other hand, longer dsRNAs may contain different regions of high silencing efficiencies, thus increasing overall efficiency of the process.

### 3.3. dsRNA Production: Increasing Quantities and Lowering Costs

In laboratory conditions, dsRNA is often synthesized using in vitro transcription kits, such as MEGA script^®^RNAi kit (Thermo Fisher Scientific, Waltham, MA, USA) and others. However, for field trials, this approach would be unacceptable due to the high costs of production (~USD 700/mg). Chemical synthesis allows large quantitates of dsRNA to be obtained but it is also very expensive with the cost increasing in accordance with the length of molecule [132]. A possible alternative is using large-scale in vivo production techniques based on genetically engineered bacteria, such as *Escherichia coli* [133]. During the last decades, the yields of dsRNA obtained by *E. coli* have significantly increased [134] mainly due to the modification of growing and nutrition conditions and development of new expressing vectors and host strains. Another principal parameter is what extraction and purification methods are used. Recent studies [135,136] showed that using cheap and highly efficient protocols for dsRNA extraction could drastically increase the total dsRNA yield and reduce its cost. Verdonckt and Broeck [137] applied a well-known system of *E. coli* HT115 strain transformed with the L4440 expressing vector to optimize the extraction and purification protocol and to show that extracted dsRNAs are not cytotoxic. The estimated price of dsRNA, obtained using optimized protocol that includes heating pre-treatment and LiCl precipitation is 43.2 EUR/mg. Apart from *E. coli*, other microorganisms can be applied to produce high quantities of dsRNA. For instance, Niehl et al. [138] developed a stable and accurate in vivo dsRNA replication system based on *Pseudomonas syringae* modified with components of phi6 bacteriophage replication complex. The risks of using bacteria to produce dsRNA for plant protection are their potential release to the environment and impact on animal and human health. As an alternative, dsRNA can be produced in other organisms, which are considered safe for human health, such as *Yarrowia lipolytica* [139] or *Saccharomyces cerevisiae* [140]. In vivo-produced dsRNAs have been successfully used for plant protection against viral [141], fungal [142] and insect [143] pathogens. Another promising way is the use of cell-free systems, able to produce dsRNA, characterized by high yield and extremely low cost (as little as USD 0.5/g, [144]).

### 3.4. Improving Host Plants’ dsRNA Uptake

An obvious problem that must be addressed for successful SIGS application in the field is dsRNA uptake by a host plant. Several factors, including dsRNA stability on the plant surface, density and behavior of stomata and specific features of waxy cuticle may significantly influence the uptake efficiency [145]. Among the approaches applied to facilitate the uptake process are using surfactants, high-pressure spraying, abrasion and stomata flooding [145,146,147]. In theory, after overcoming the cuticle barrier, a dsRNA molecule should bypass the apoplast–symplast system, entering the plant cell to be processed by plant RNAi machinery. However, in the case of toxigenic fungi such as *F. graminearum*, pathogens mainly uptake unprocessed dsRNAs and digest them by its own RNAi machinery [76]. Therefore, the development of methods improving dsRNA movement via plant vasculature is needed. One of these approaches, based on the use of carrier molecules and different types of formulations, is considered in detail in the next section.

### 3.5. dsRNA Formulation: Improving Delivery Efficiency and Overcoming Degradation

In the majority of studies performed so far, dsRNAs are delivered to a mycotoxigenic fungus in a naked, unformulated variant, characterized by a restricted lifetime and efficiency, especially in planta. One of the most promising approaches to solve these problems is using nanoparticles as dsRNA carriers. An example of the successful application of carriers to protect plants against mycotoxin producers is the work of Power et al. [127]. The authors used 0.6 µm gold particles to coat both dsRNA and dsDNA and for their delivery to *A. flavus*-infected peanut plants by particle bombardment. This approach has proven effective, but it is technically sophisticated and hard to be widely applied. On the other hand, nanocarriers have been actively used in RNAi-based plant protection against different pathogens. Positively charged layered double hydroxide (LDH) nanosheets, also called ‘BioClay’, were among the first nanomaterials successfully used in plant pathology. Mitter et al. [148] applied LDH to protect cowpea and tobacco against the cucumber mosaic virus (CMV) and pepper mild mottle virus (PMMoV). The authors declared that LDH-formulated dsRNA provided up to 20-days protection of plants against viruses, compared to 5 days of protection using naked dsRNA. Mosa and Youssef [149] used LDH nanosheets to protect dsRNAs targeted to essential genes of *F. oxysporum* f. sp. *radicis-lycopersici* causing crown and root rots in tomatoes and showed that this technology provided plant protection for at least 60 days. Chen et al. [150] declared that using dsRNAs loaded on LDH nanosheet significantly improved the efficiency of management maize and tobacco infections caused by *Rhizoctonia solani*. Another material, widely used to protect dsRNA and to deliver it to target cells is chitosan (poly β-1,4-D-glucosamine, [151]). Chitosan is a biocompatible material, characterized by low toxicity, inexpensive production and a wide range of modifications [152]. Chitosan-based nanoparticles have been used to protect plants against insects, such as cotton bollworm (*Helicoverpa armigera*, [153]) and stem borer (*Chilo supressalis*, [154]). In a recent study [155], six different types of nanoparticles—chitosan (CS), polyethyleneimine (PEI), protamine, carbon quantum dot (CQD), polyamidoamine (PAMAM) and chitosan/star polycation complex (CSC)—were used as carriers for dsRNAs targeting two genes related to the virulence of *Rhizoctonia solani*. All the nanoparticles could assemble with dsRNA, but CSC also enhanced the stability of the molecule, reduced pathogen infection on rice and prolonged the protection time up to 20 days. Based on the result, the authors declared that SIGS-based CSC-dsRNA delivery systems have the potential to be commercialized and used for plant fungal disease management in the field.

Qiao et al. [156] proposed to use artificial nanovesicles for dsRNA delivery to combat *Botrytis cinerea* on tomato and grape fruit leaves. It was shown that AVs application extended the time of dsRNA-based protection to 10 days on tomato and grape fruits and to 21 days on grape leaves. Another recent study demonstrated that *E. coli*-derived anucleated minicells can be applied for dsRNA production, encapsulation and delivery [157]. In this work, the authors obtained an *E. coli* mutant producing a large number of minicells with compromised RNase-III activity and transformed it with vectors expressing chitin synthase class III (Chs3a, Chs3b) and DICER-like proteins (DCL1 and DCL2) genes of *Botryotinia fuckeliana*. The production of minicell-encapsulated dsRNA in bioreactors was about 100 mg/L. Topical spray application of dsRNA packaged in minicells on strawberry 1 h before inoculation of *B. fuckeliana* led to complete inhibition of fungal growth and disease development (while naked dsRNA was applied, a minimal fungal growth was observed). It was shown that the use of minicell-encapsulated dsRNAs prolonged the strawberry protection against *B. fuckeliana* up to 12 days. Niño-Sánchez et al. [158] proposed to use non-pathogenic bacteria as a system to deliver RNAi to fungal cells as an alternative to HIGS and SIGS assays. In the study, the RNAse III null-mutant strain of *E. coli* HT115 was transformed with two plasmid vectors able to express dsRNA against the *AFLC* gene involved in aflatoxin synthesis in the *A. flavus* and *BcSaS1* genes, responsible for virulence in *Botrytis cinerea*. The authors demonstrated that under in vitro conditions, exposure to both living bacteria or whole-cell lysates induced silencing of the target genes, leading to inhibiting aflatoxins production and mycelial growth, respectively.

Generally, different prospective types of potential nanocarriers are thoroughly discussed in several reviews [159,160,161]. The use of nanoparticle-based approaches significantly improve dsRNA stability, enhance its uptake by plant and target organisms, and prolong the time of protection. At the same time, it should be noted that several types of nanoparticles may be linked to higher toxicity in plants, animals and soil microflora. Moreover, they potentially negatively impact human health: for example, inhaling nanoparticles may result in lung inflammation and heart problems, and their ability to overcome the blood–brain barrier may lead to delivering toxic compounds to the brain and central nervous system [162,163]. Therefore, extensive studies on the safety of nanoparticles and estimation of corresponding risks are of great importance.

### 3.6. Nanoparticle-Free Approaches for RNAi-Based Systems

Recently, techniques of increasing the stability of dsRNA and its delivery efficiency avoiding the use of nanoparticles are being developed. Such an innovative approach was proposed by Singewar and Fladung [164] and called ‘mycorrhiza-mediated dsRNA delivery method’. In this approach, stressed plants are inoculated with mycorrhiza cultures carrying dsRNA (obtained through the direct uptake of dsRNA or by transformation) which targets a specific pathogen’s gene. In the mycorrhizal system, the nutritional exchange takes place between fungal and plant root cell walls. The fungus is able to alter the plant’s cell wall and cause beneficial invasion, helping to transfer dsRNA to the plant where the molecule is further transported to the stem and leaves through the vascular system. Therefore, in theory, this approach can provide a double benefit, including traditional advantages of mycorrhiza together with dsRNA-based silencing of pathogens. Another promising technique is chemical modifications of dsRNA [165,166]. The therapeutic meaning of chemically modified short RNAs is well known in medicine [167], but their potential to be used in agricultural systems is still to be elucidated. In one of the first studies, Howard et al. [168] demonstrated that phosphorothionate- and 2′-fluoro-modified long dsRNAs were able to induce RNAi in *Drosophila* cell lines, live green stink bug (*Nezara viridula*) nymphs and live western corn rootworm (*Diabrotica virgifera virgifera*) larvae with higher efficacy compared to unmodified dsRNAs. In live insects, treatment by the modified molecules resulted in mortality in the latter two species. In addition, the modified dsRNAs were stable to environmental nucleases, including those present in soil and stink bug saliva. These results indicate the possibility of using chemically modified long dsRNAs for RNAi-based plant protection. Further studies on the different pathogens, including fungi, with the use of different modifications are required to develop RNAi-based control protocols.

## 4. Conclusions and Perspectives

Since mycotoxins represent one of the most significant risks for agriculture and food safety, methods of reliable, eco-friendly and cheap control of mycotoxigenic fungi are of high importance. RNAi-based techniques have been extensively used for different scientific and applied purposes. Both HIGS and SIGS have proven effective to protect agricultural plants against producers of key mycotoxins and have the great potential to replace traditional fungicides. However, the ultimate goal, undoubtedly, should be the implementation of this approach in agricultural practice and the development of RNAi-based biopesticides. To meet this challenge, the following issues should be addressed: (i) expansion of the list of fungal species and target genes tested; (ii) development and improvement of algorithms for rational dsRNA design and identification of potential off-targets; (iii) elucidating the optimal dsRNA parameters (concentration, molecule length) for in vitro and *in planta* applications; (iv) improvement of techniques for cost-effective and large-scale dsRNA production; (v) developing the methods of efficient dsRNA protection and targeted delivery to a pathogen. Besides solving these practical tasks, further basic research of fungal RNAi machinery, mechanisms of RNA uptake, transport in plants and between host and pathogen and factors influencing these processes are needed. Little is known about the persistence, durability and side effects of dsRNAs on host plants. In addition, implementation of RNAi-based approaches in agricultural practice will require consideration of the biosafety of new dsRNA pesticides. Finally, the current legislations should be adopted to approve the RNAi-based methods and products. Solving these problems will pave the way for these products to successful field trials and entering the market.

## Data Availability

Data are contained within the article.
